# The adverse effects of novel coronavirus on diabetic foot patients: A protocol for systematic review and meta analysis

**DOI:** 10.1097/MD.0000000000022758

**Published:** 2020-10-23

**Authors:** Dongqiong Chen, Hui Zhou, Yan Yang, Yuan Zhang, Chunguang Xie

**Affiliations:** Hospital of Chengdu University of Traditional Chinese Medicine, Chengdu, Sichuan Province, China.

**Keywords:** coronavirus disease -19, diabetic foot, protocol, systematic review and meta-analysis

## Abstract

**Background:**

Since the outbreak of novel coronavirus in 2019, the number of new coronavirus infections worldwide has been increasing, there is no effective treatment or vaccine. Novel coronavirus infection is closely related todiabetes, the mortality of diabetes with novel coronavirus pneumonia is significantly higher than that of non diabetic with novel coronavirus pneumonia, Diabetic foot is one of the common and serious complications of diabetes, however, no systematic study on novel coronavirus pneumonia adverse effects on diabetic foot has been found at home and abroad, however, this is a problem that can not be ignored.

**Methods:**

We will search each database from the built-in until April 2021. The English literature mainly searches Cochrane Library, PubMed, EMBASE, and Web of Science, while the Chinese literature comes from CNKI, CBM, VIP, and Wangfang database. Simultaneously we will retrieval clinical registration tests and grey literatures, and he researches related to the adverse effects of novel coronavirus on diabetic foot were collected, The 2 researchers worked independently on literature selection, data extraction, and quality assessment. The dichotomous data is represented by relative risk, and the continuous is expressed by mean difference or standard mean difference, eventually the data is synthesized using a fifixed effect model or a random effect model depending on whether or not heterogeneity exists. The primary outcome was clinical response rate, C-reactive protein and procalcitonin. Secondary outcomes are mainly including mortality, amputation rate, wound healing time and nerve conduction velocity. Finally, meta-analysis was conducted by RevMan software version 5.3.

**Results:**

The results of our research will be published in a peer-reviewed journal.

**INPLASY registration number:**

202080113

## 1 Introduction

Since the outbreak of coronavirus in December 2019, because of its high infectivity, high pathogenicity and high mortality, it has caused a global pandemic.^[1–3]^ According to official data,^[4]^ As of 24:00, August 22, 2020, Beijing time, The number of confirmed cases has exceeded 10 million and the number of deaths has exceeded 800 thousand, Novel coronavirus pneumonia poses a great threat to global human health. Diabetes is one of the three major chronic non communicable diseases threatening human health, According to the latest data of the International Diabetes Federation, by the end of 2019, the number of diabetes patients in the world has exceeded 463 million.^[5]^ Diabetic foot is one of the common and serious complications of diabetes. The global prevalence of diabetic foot ulcer is 6.3%.^[6]^ Diabetic patients are more likely to be infected with new coronavirus due to hyperglycemia, chronic inflammation, microcirculation damage and other factors, and the severe and mortality rate is higher,^[7–9]^ Novel coronavirus infection is associated with angiotensin-converting enzyme 2 (ACE2) and may promote interpersonal transmission.^[10]^ Diabetes can up regulate the expression of ace in lung and ACE2 in pancreas,^[11]^ novel coronavirus infection may interact with diabetes, leading to severe infection and death in patients with new coronavirus infection. At present, the novel coronavirus pneumonia patients with diabetes are mostly studied in terms of their infection rate and mortality rate, however, the effect of new coronavirus on adverse reactions in patients with diabetic foot is not clear, this is a problem that cannot be ignored.

## 2 Methods

### 2.1 Protocol registration

The systematic review protocol has been registered on the inplasy website as INPLASY202080113 (https://inplasy.com). It is reported following the guidelines of Cochrane Handbook for Systematic Reviews of Interventions and the Preferred Reporting Items for Systematic Reviews and Meta-analysis Protocol (PRISM).^[15]^ We will update our protocol for any changes in the entire research process if needed.

### 2.2 Inclusion criteria

Novel coronavirus pneumonia with diabetic foot patient will be included in our study. There are no restrictions on the region, gender, and age of patients.

#### 2.2.1 Study design

This study only selected the adverse effects of novel coronavirus on diabetic foot patients published in both Chinese and English. However, animal experiments, reviews, case reports, and non-randomized controlled trials are excluded.

#### 2.2.2 Participants

Novel coronavirus pneumonia with diabetic foot will be included in our study. There are no restrictions on the region, gender, and age of patients.

#### 2.2.3 Intervention

This study will investigate a comparison of patients with diabetic foot with coronavirus disease -19 (COVID-19) and non-diabetic foot with COVID-19, According to whether diabetic foot is combined, they are divided into diabetic foot group (trail) and non diabetic footgroup (comparison). Patients who novel coronavirus pneumonia without foot disease will be excluded.

#### 2.2.4 Outcomes

The primary outcome was clinical response rate, C-reactive protein and procalcitonin. Secondary outcomes are mainly including mortality, amputation rate, wound healing time and nerve conduction velocity

### 2.3 Search methods

#### 2.3.1 Electronic searches

We will retrieve each database from the built-in until May 2021. The English literature mainly searches Cochrane Library, PubMed, EMBASE, and Web of Science. While the Chinese literature comes from CNKI, CBM, VIP, and Wangfang database. We adopt the combination of heading terms and free words as search strategy which decided by all the reviewers. Search terms: diabetes, diabetic foot, diabetic foot disease, diabetic foot ulcer, COVID-19, novel coronavirus, novel coronavirus infection. We will simply present the search process of the Cochrane library (Table 1). Adjusting different search methods according to different Chinese and English databases.

**Table 1:**
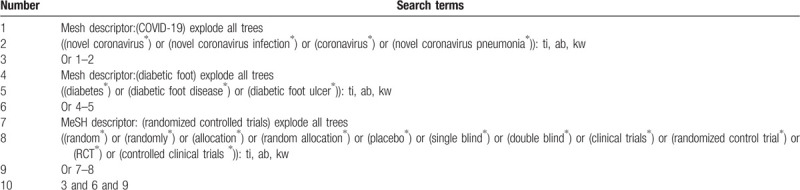
Cochrane library search strategy (Table 1 Example of Cochrane search strategy).

#### 2.3.2 Searching other resources

At the same time, we will retrieve other resources to complete the defificiencies of the electronic databases, mainly searching for the clinical trial registries and grey literature about for novel coronavirus pneumonia with diabetic foot on the corresponding website.

### 2.4 Data collection and analysis

#### 2.4.1 Selection of studies

**Figure 1:**
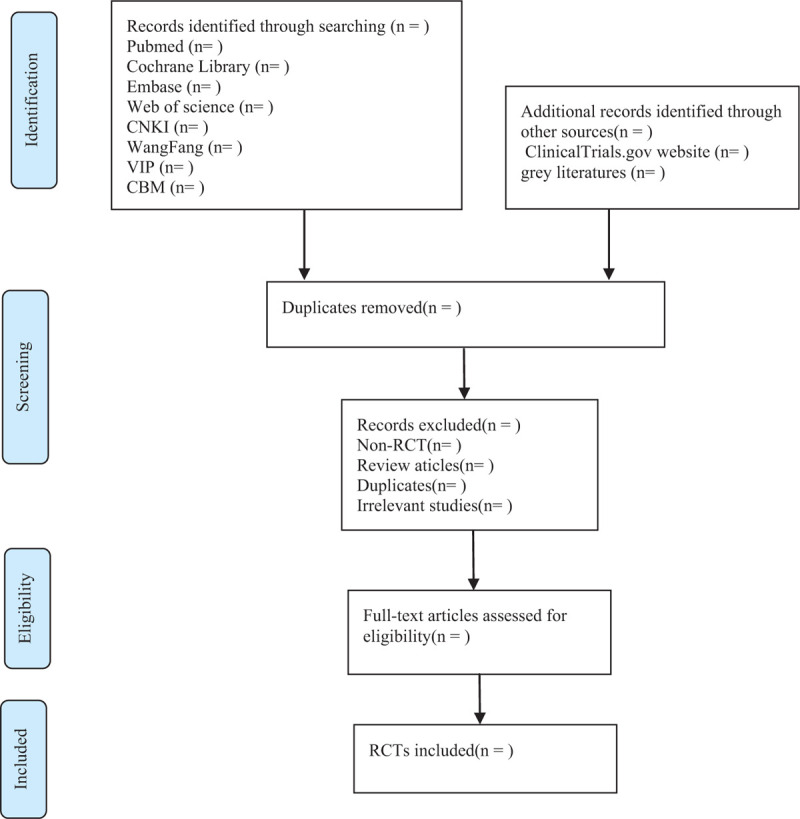
Flow chart of the study selection.

Import all literatures that meet the requirements into Endnote X8 software. First of all, two independent reviewers initially screened the literatures that did not meet the pre-established standards of the study by reading the title and abstract. Second, download the remaining literatures and read the full text carefully to further decide whether to include or not. Finally, the results were cross-checked repeatedly by reviewers. If there is a disagreement in the above process, we can reach an agreement by discussing between both reviewers or seek a third party's opinion. Flow chart of the study selection (Fig. 1) will be used to show the screening process of the study.

#### 2.4.2 Data extraction and management

According to the characteristics of the study, we prepare an excel form for data collection before data extraction. Outcome indicators for eligible studies were independently extracted and fifilled in the data extraction form by two reviewers. The main data extracted are as follows: title, author, year, fund source, sample size, age, sex, duration of disease, interventions, outcome measures, adverse reactions, etc. If there are something unclear, you can not hesitate to contact authors of more detailed information. The above information was fifinally cross-checked by 2 reviewers.

#### 2.4.3 Assessment of risk of bias in included studies

The quality assessment of randomized controlled trials adopts the risk of bias (ROB) assessment tool provided by the Cochrane Handbook. The following seven items, such as random sequence generation, allocation concealment, blinding of participants and personnel, blinding of outcome assessment, incomplete outcome data, selective outcome reporting, and other bias, are evaluated by three grades of “low bias,” “high bias,” and “unclear bias.” The discrepancies will get a consistent conclusion by discussing between both reviewers or seeking the third-party consultation.

#### 2.4.4 Data analysis

Different evaluation methods are selected according to the different effificacy indicators. For the dichoto mous data, we will choose the effect scale indicator relative risk with 95% confifidence interval to represent. While the continuous data is expressed as mean difference or standardized mean difference with 95% confidence interval depending on whether the measurement scale is consistent or not. Review different causes of heterogeneity. If a meta-analysis cannot be performed, it will be replaced by a general descriptive analysis.

#### 2.4.5 Investigation of heterogeneity

If there is substantial heterogeneity between studies, then we will conduct subgroup analysis to explore the heterogeneity. To avoid post hoc analysis, the subgroup analysis will be conducted according to 3 hypotheses: race, course of diabetes, glucose level. To further improve the reliability of subgroup analysis, we will evaluate the credibility of our subgroup analysis according to the guidance for credible subgroup analysis. If there are enough studies included, then meta-regression will be conducted to further explore the heterogeneity. Those subgroup effects that occur simultaneously in subgroup analysis and regression analysis will be considered credible.

#### 2.4.6 sensitivity analysis

Draw funnel chart for the adverse effects indicators of diabetes and non diabetes COVID-19 patients. To ensure the stability of the results, we will conduct sensitivity analysis of the results by excluding each of the studies included in the analysis one by one, then re-analyzing the results, and comparing the differences between the re-obtained results and the original results. In this way, we will be able to assess the impact of individual studies on overall outcomes and their robustness.

#### 2.4.7 Reporting bias assessment

The integrity of the studies is an important factor affecting the accuracy of the results and conclusions of meta-analysis. The integrity of the included studies is mainly measured by reporting bias, of which publication bias is the most common. Therefore, this study will identify report bias by publication bias assessment. A funnel plot will be drawn to investigate the publication bias. Funnel plot will be asymmetric when publication bias exists, such as when research with small sample and no statistically significant results are not published. The more obvious the asymmetry of funnel plot is, the more likely there is publication bias^[22]^ And then Egger test will be conducted for statistical assessment the publication bias. The publication bias is considered to exist if *P* < .05^[23].^

#### 2.4.8 Patient and public involvement

No patients or public will participate in the study.

#### 2.4.9 Ethics and dissemination

Since confidential patient data will not be involved in this study, formal ethics approval is not required. The frame- work of the Preferred Reporting Items for Systematic Reviews and Meta-Analysis Protocol statements for NMA will be applied to guide review authors to perform this study. The results will be disseminated by a peer-reviewed publication.

## 3 Discussion

Novel coronavirus pneumonia (COVID-19) is still spreading, no specific drugs or vaccines against the virus have been found, the treatment is basically symptomatic treatment.^[12–14]^ In patients with severe acute respiratory syndrome coronavirus infection and Middle East respiratory syndrome coronavirus infection, the proportion of patients with diabetes mellitus is high, and diabetes mellitus is the risk factor of death.^[15,16]^ Some studies have shown that, the CRP and PCT of patients with new crown and diabetes mellitus were significantly higher than those of patients without diabetes mellitus, and the immune system was greatly inhibited.^[17–19]^ Thus, the novel coronavirus can affect the clinical cure rate of diabetic foot patients, delay the healing time, increase the amputation rate and mortality rate.

## Author contributions

**Conceptualization**: Dongqiong Chen, Hui Zhou, Chunguang Xie. Data curation: Yuan Zhang.

**Formal analysis**: Dongqiong Chen, Hui Zhou.

**Funding acquisition**: Chunguang Xie.

**Investigation**: Yuan Zhang.

**Methodology**: Dongqiong Chen, Yan Yang, Hui Zhou.

**Project administration**: Chunguang Xie.

**Resources**: Dongqiong Chen, chunguang Xie.

**Software**: Dongqiong Chen, Hui Zhou.

**Supervision**: Yuan Zhang.

**Writing – original draft**: Dongqiong Chen.

**Writing – review & editing**: Chunguang Xie.
